# ‘We work together as a group’: implications of jigsaw cooperative learning

**DOI:** 10.1186/s12909-023-04734-y

**Published:** 2023-10-06

**Authors:** Ashok Kumar Jeppu, Kavitha Ashok Kumar, Ahsan Sethi

**Affiliations:** 1 International Medical School, Management and Science University, University Drive, Section 13, Shah Alam, Malaysia; 2 QU Health, Qatar University, Doha, Qatar

**Keywords:** Cooperative learning, Jigsaw, Medical students, Social skills

## Abstract

**Background:**

Modern clinical practice increasingly relies on collaborative, cooperative and team-based approaches for effective patient care. Recently, Jigsaw cooperative learning has gained attention in medical education. There is a need for studies in Southeast Asian context to establish its effectives in developing various core competencies expected of health professionals such as interpersonal, communication, collaborative, and teamwork skills. This current study explores the impact of using Jigsaw Cooperative Learning on undergraduate medical students.

**Method:**

An explanatory mixed method research design was carried out on first year medical students at a private university in Malaysia. In Phase I, a survey was conducted to explore the effectiveness of jigsaw learning. Descriptive and inferential statistics were calculated using SPSS. In Phase II, a focus group interview was conducted to explore their in-depth experiences. Qualitative data were thematically analysed.

**Results:**

Fifty-seven students participated in the survey and seven students took part in the focus group interview. Quantitative data analysis showed a statistically significant improvement in the student’s individual accountability, promotive interaction, positive interdependence, interpersonal skill, communication skill, teamwork skill, critical thinking and consensus building after jigsaw learning sessions. Qualitative data explained their experiences in-depth.

**Conclusion:**

Jigsaw cooperative learning improves collaboration, communication, cooperation and critical thinking among the undergraduate medical students. Educators should use jigsaw learning methods to encourage effective collaboration and team working. Future studies should explore the effectiveness of the jigsaw cooperative learning technique in promoting interprofessional collaboration in the workplace.

## Introduction

Modern clinical practice increasingly relies on collaborative, cooperative and team-based approaches for effective patient care [1]. Future health professionals should learn and practice these core competencies from an early stage of medical training [2, 3]. The responsibility of transforming the “competitive” students into “collaborators” rests on the health professions educators. Regulatory bodies in medical education also emphasise the need to develop interpersonal, communication, collaborative and teamwork skills among medical students [4, 5]. Achievement of these competencies have implications for students learning experience, their professional identity formation and in turn the quality of healthcare provided [6].

In medical schools, student-centred learning is often characterised by small group work. The students are expected to collaborate and learn together in these small groups. However, merely assigning tasks in small groups does not automatically lead to effective collaborative learning, which continues to be a challenge [7]. There is a potential for students to experience unequal participation, ineffective communication, conflicts and difficult members (e.g., shy/disruptive/dominant) in their groups [8–10]. The instructor must be cognizant of how best to facilitate effective collaborative learning environments.

Cooperative learning (CL) is a well-structured and carefully planned learning strategy, used to facilitate a sustained learning group with interdependent members working towards a specific academic goal under guidance [11]. This strategy is extensively used in high schools, contributing to the majority of published literature [12]. This is slightly different from collaborative learning, which is an umbrella term for learners working together on a task as a group. In cooperative learning, the group members have a predefined role, set of tasks and goals. They must master their part of the work and share this information with the group for collective understanding. The division of tasks assures accountability, and all members are responsible for the learning of theirs as well other members of the group [13]. In this article we use the term cooperative learning, as the jigsaw method involves structured group work with all characteristics of cooperative learning.

Jigsaw is a cooperative learning strategy [14, 15], where each student of a “Home” group (a small group of students) chooses or is allocated a sub-topic related to the main topic to research. All the students from different “Home” groups with the same sub-topic assemble together to form an “Expert” group, where they research, discuss, and specialize in the given sub-topic. After mastering the sub-topic, the student returns from the “Expert” group” to the "Home" group and teach their allocated sub-topics to ensure holistic understanding of the main topic to the group members during the activity. Therefore, each student in the “Home” group serves as a piece of the topic's puzzle. Each part must work together and fit in perfectly to complete the whole jigsaw puzzle. This involves active and social learning by the virtue of peer interaction [16].

Over the last few years, there has been a growing interest in using the Jigsaw Cooperative Learning strategy in higher education [15, 17]. Previous studies in medical education, have explored the effectiveness of Jigsaw Cooperative Learning quantitatively [14, 18–20]. Most of the previous studies used structured questionnaires and some added open-ended questions to supplement the quantitative data. There is a need for rigorous in-depth studies on establishing the effectiveness of Jigsaw Cooperative Learning in developing various core competencies expected of health professionals such as interpersonal, communication, collaborative, and teamwork skills [21, 22]. Moreover, there is paucity of literature in the Southeast Asian countries with strong social hierarchies among students [23]. Such hierarchies make it difficult for students to fully embrace a student-centred learning, where they are expected to take more control of their learning and actively participate in small groups. The Jigsaw Cooperative Learning may help narrowing the wide social distance in pursuit of a more equitable and inclusive educational environments [24]. This current study explores the impact of using jigsaw cooperative learning (JCL) on undergraduate medical students at a private university in Malaysia, which includes student from various ethnic groups like Malay, Indian and Chinese.

### Methods

#### Study design

This current study employed an explanatory mixed method research design. In phase I, a quantitative survey was conducted to measure the impact of Jigsaw Cooperative Learning on undergraduate medical students. In phase II, focus group interview were conducted to complement, clarify and extend the quantitative results. Ethical approval was obtained from the University Ethics Committee (MSU-RMC-02/FR01/01/L1/014).

#### Questionnaire and interview guide

A self-reported questionnaire was developed based on the literature review [25, 26]. Using a four-point Likert scale, the questionnaire assessed students’ perception of various aspects of cooperative learning such as individual accountability, face-to-face promotive interaction, positive interdependence, interpersonal skill, communication skill, teamwork skill, critical thinking, problem solving and consensus building. The questionnaire was validated by experts (*n* = 6) and it was piloted on students (n = 40) to check for comprehension and clarity. The items had good internal consistency (Cronbach’s alpha 0.879).

A focus group interview guide was developed based on the social interdependence theory [27, 28]. According to this theory, an individual’s achievement depends on other’s actions and cooperation which is essential for cognitive growth [28, 29]. The questions explored student’s experience of jigsaw learning sessions in-depth along with its impact on their development.

#### Jigsaw Cooperative Learning (JCL)

**Fig. 1:**
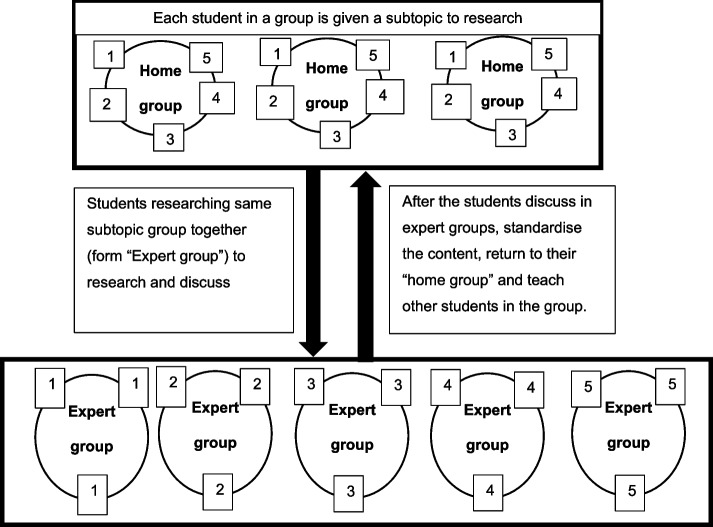
Jigsaw cooperative learning technique

A total of seven JCL sessions of two hours duration each were carried out. Participants were undergraduate medical students of April 2020 cohort (*n* = 63) in their first semester at a private university in Malaysia. A large group of students were divided into smaller heterogeneous (Home) groups based on their academic performance, gender and ethnicity. The facilitator introduced the topic and distributed different subtopics to each member of the home group. Students from different “Home” groups researching the same sub-topic regrouped in an “Expert” group to research, discuss, synthesize, and prepared the learning material. Later, students from each “Expert” group returned to their "Home" groups, where they taught their sub-topics to their group (Fig. 1). Finally, students presented their group work to the facilitator and the sessions concluded with reflection and feedback.

#### Data collection

Students’ perception before and after the JCL was collected using questionnaire. The questionnaire also helped recruit participants for the focus group interview. Among those who consented, a purposive maximum variation sample of seven students were invited for focus group interview. The interview was audio recorded and transcribed verbatim.

#### Data analysis

The quantitative data was analysed using SPSS.v.26. Descriptive and inferential statistics were calculated. A paired sample t-test was used to compare mean scores at the beginning and end of the cooperative learning session. The qualitative data were thematically analysed [30]. Each transcript was read independently and coded by both the authors (AKJ and AS). The initial codes with similar patterns were categorised and refined into sub-themes and themes through continuous deliberation and memo-writing.

## Results

Ninety percent of students responded to the survey. The participants were predominantly females and from Malay ethnicity. The focus group interview included seven students of different ethnicity and gender (Table 1).

**Table 1:** Participant characteristics

Characteristics		Frequency (N)	
		Survey	Focus group interview
Gender	Male	22	4
	Female	35	3
Age	< 20 Years	27	3
	≥ 20 years	30	4
Ethnicity	Malay	27	2
	Indian	15	4
	Other	15	1

The quantitative data analysis showed that the participants significantly improved on most of the domains including their individual accountability with regards to submitting their assignments and doing the work entrusted to the best of their ability. Their interpersonal skills and interdependence i.e., group members assisting each other while solving problems had also increased significantly. Their communication skill, teamwork skill, critical thinking and consensus building also improved significantly (Table 2).

**Table 2:** Impact of cooperative learning

Elements of CL	Statements	Before CL		After CL		*P*-value
		Mean	SD	Mean	SD	
Individual Accountability	I attend all my classes and I am on time	3.47	.68	3.61	.52	.059
	I dress and behave professionally	3.42	.62	3.56	.53	.059
	I prepare and submit my assignments on time	3.46	.60	3.58	.49	.018*
	I do the work entrusted to me to the best of my ability during class activities	3.25	.63	3.42	.53	.024*
Interpersonal skill	I find interacting and working with my peers motivating	2.93	.72	3.35	.58	.000*
	I interact with my peers to obtain a deeper understanding of the subject	2.84	.75	3.35	.64	.000*
	I take into account the knowledge of my peers	3.19	.58	3.42	.53	.000*
Positive Interdependence	We assist each other while solving problems during the teaching learning session	3.00	.68	3.35	.55	.001*
	We depend on each other more than on the tutor/lecturers for learning	2.56	.80	2.72	.88	.060*
Communication skill	I listen to and respect the ideas of others	3.33	.57	3.54	.53	.002*
	I am able to clearly and effectively express my views	3.04	.73	3.21	.64	.040*
Teamwork Skill	I get along easily with my colleagues and get their cooperation	2.96	.73	3.25	.66	.003*
	I contribute effectively to achieve the common goal during the learning activity	3.16	.62	3.35	.55	.033*
Critical Thinking	I am able to identify critical concepts of the topics I learnt	2.89	.67	3.26	.55	.000*
	I am able to assess the credibility of the statements made by my peers during discussion	2.96	.62	3.26	.58	.000*
	I am able to use concepts and evidence to justify my thinking and analysis	2.96	.62	3.23	.46	.001*
Consensus Building	I can make effective decisions together with my peers	2.96	.65	3.33	.54	.000*

^*^p-value < 0.05

The findings of the qualitative study are reported in Table 3. After JCL, the students reported greater ‘understanding’ of the subject (Excerpt-1). Teaching peers helped them to ‘remember’ the content (Excerpt-2) and to become ‘knowledgeable’ (Excerpt-3). It helped them stay ‘committed’ (Excerpt-4) to gather information and take ‘responsibility’ (Excerpt-5) for their peers’ learning. It encouraged ‘exchange of ideas’ and promoted ‘guidance’ among group members (Excerpt-6&7). Students felt’motivated’ to research extensively and be well prepared as other group members relied on them for information (Excerpt-8), which they perceived as ‘eustress’ (Excerpt-9). Fruitful discussion not only led to ‘exchange of knowledge’ (Excerpt-10), but also lead to development of mutual ‘trust’ (Excerpt-11), respect for others’ opinions (Excerpt-12&13) and helped to ‘think critically’ to acquire a deeper understanding (Excerpt-27). Students seeking help from their peers to derive ‘solutions to problems’ (Excerpt-17). lead to “interdependency” (Excerpt-16). The students realized that each member had an equal chance to contribute to a ‘better outcome’ (Excerpt-14) ‘without conflicts’ (Excerpt-28). During the discussions, their peers ‘shared their work’(Excerpt-26), provided ‘support’ (Excerpt-15), which facilitated them to ‘work together’ (Excerpt-25). with ‘cooperation’ (Excerpt-24). The ‘language’ used during the group discussion, was simple and easy to comprehend (Excerpt-18). They learnt the importance of ‘listening’ to others’ point of view (Excerpt-20). It helped students to ‘explain better’, improve their ‘presentation skill’ (Excerpt-19), ‘boosted their ‘confidence’ (Excerpt-22), ‘overcame their shyness’, and became more outspoken (Excerpt-21&23).

**Table 3:** Impact of cooperative learning

Promote Learning	Understanding	**Excerpt-1:** Really good, because we have two or three steps on improving our understanding … regarding the subject (MaleM#2)^a^
	Remembering	**Excerpt-2:** When we present … to our friend we will remember it longer (FemaleM#7)
	Knowledgeable	**Excerpt-3:** It enabled us on how to be like knowledgeable (FemaleI#6)
Individual accountability	Commitment	**Excerpt-4:** I will try my best to gather as much information as possible, and I like to share that with my friends (FemaleI#4**)**
	Responsibility	**Excerpt-5:** We feel like it's our responsibility to teach our friends (FemaleM#7)
Interpersonal skill	Exchange of ideas	**Excerpt-6:** Discussions very good because we really exchange our idea as much as we can (MaleM#2)
	Guidance	**Excerpt-7:** Our group members will be like are always aware of what is going on and we have understanding more about the topic (MaleI#3)
	Motivation	**Excerpt-8:** Like how that tempting sort of forces you to study because if you are assigned to that certain thing all other teammates are expecting on you for that information (MaleI#1)
	Eustress	**Excerpt-9:** Cooperative learning might be like stressful… have to find a lot of information and then put more effort, I think it's kind of like a good stress because it will drive you to find more information (FemaleI#6)
	Knowledge sharing	**Excerpt-10:** We can share our knowledge on particular area with others (MaleI#3)
	Trust	**Excerpt-11:** I also understand that our classmates can also be referred to, when we have a certain issue (MaleI#1)
	Respect for others opinion and knowledge	**Excerpt-12: **Prior to that I thought I also have no idea how my friend would have any idea, but you know after the after this cooperative learning I understood that my friends also powerful, you can refer to for knowledge (MaleI#1)
	Equality	**Excerpt-13:** With this cooperative learning I understood that, you know, like all my classmates are equally talented, they have equal amount of knowledge as me as well (MaleI#1)
Positive interdependence	Contribution for better outcome	**Excerpt-14:** Each of us get a chance to exchange our idea between us, every one of us give our opinion building the most perfect answer for that question (MaleM#2**)**
	Support	**Excerpt-15:** Whenever we are out of track in discussion our group members who are aware of that will bring back to track (MaleI#3)
	Interdependence	**Excerpt-16:** I started referring to my friends, then if they don't know I refer to my lecturers (MaleI#1)
	Problem solving in a team	**Excerpt-17:** In the cooperative session, whenever we are unable to find an answer if let's say for one question, I don't know the answer and then we work together as a group, we ended up finding a fine one solution for the problem (FemaleI#4)
Communication skill	Language	**Excerpt-18:** Whenever it's cooperative learning it can build our communication skill, as the suitable language that we understand we'll be using (MaleI#3)
	Presentation skill	**Excerpt-19:** Can improve in terms of the presentation skills (MaleM#2)
	Listening to others	**Excerpt-20:** I understood that sometimes we must allow other people to speak up. We can continuously be giving our opinions, because they also have important things that they may bring to the table (MaleI#1)
	Explain better	**Excerpt-21:** I learned on how to speak out, how to explain to people better (FemaleM#7)
	Confidence	**Excerpt-22:** We have a good self-confidence so that we can explain to another person like smoothly (MaleM#2**)**
	Overcoming shyness	**Excerpt-23:** Some of the people are shy to voice their opinion, and maybe no one wants to be the first person to begin the answer, and this may break long time best thing happened for me (MaleO#5)
Teamwork	Cooperation	**Excerpt-24:** I overcome the like the competition in my group mates is by I learned in terms of how to work as team how to tackle them how to make them more interested towards the topic that we are learning (FemaleM#7)
	Work together	**Excerpt-25:** Very helpful, especially in my group as time goes by, it made our group more improve in terms of working together (FemaleM#7)
	Share the work	**Excerpt-26:** I can understand more because each and everyone us we when divide and do the work (FemaleI#4)
Critical thinking		**Excerpt-27:** I won't be thinking that type of question, but my friends who asked me like why it is happening…why not like this… it would…trigger me more…acquire and find more knowledge (MaleI#3)
Consensus building		**Excerpt-28:** We also need to be ready, listen to other people's opinion also and we also can learn the way how we will do the discussion, so that we won't have a fight during the discussion (MaleO#5)

^a^The identifiers are based on the combination of gender, ethnicity (M-Malay, I-Indian, O-Others) student number

## Discussion

### Impact on students learning

Future physicians are expected to be knowledgeable and be team players with excellent social skills [3, 6, 31]. This present study showed that the Jigsaw Cooperative Learning (JCL) technique was effective in enhancing these skills. The students reported that their understanding and knowledge retention had considerably improved after the intervention. An improvement in learning after using this educational tool was also observed by Nusrath, et al. [26] and Uppal & Uppal [32]. During JCL, the learners were able to rehearse their learning which helped restructure the information and improved retention in their memory [33]. When students are exposed to peers of intellectual diversity with varied ways of looking at familiar problem, deeper learning is promoted [34]. From the social cognitive perspective, students construct their knowledge by discursive and dialogic process [35]. While from the cognitive science viewpoint, JCL helps to provide a conceptual framework to understand what is being learnt [36].

The students’ ability to identify the concepts, use evidence to justify their thinking and analyse the statements made during the discussions had improved significantly after the intervention. The cognitive conflict during the activity helped develop critical thinking skills [37]. Cooperative learning therefore can be used to teach critical thinking skills which enables learners to apply the knowledge acquired in the classroom to the workplace [38].

### Impact on students’ social skills

An improvement in individual accountability was observed in this study. It may be an outcome of students’ realisation of their responsibility for the learning of their peers. This finding was affirmed by Pateşan et al., [37] as well. A member of the group, entrusted with the task to teach a subtopic leads to enhancement of their personal as well as public accountability [14]. The sessions significantly improved understanding and motivation among the participants of this study. Active engagement [39], similar thought process among students [26], motivation to participate in the learning activities [37], self-regulated learning [40] and improved collaboration [41] could have contributed to the improved interpersonal skills of the JCL group participants. JCL boosted interdependence among students. A better bonding and rapport between the students were also reported by Bhandari et al., [14]. JCL brings about positive interdependence as students’ need to assist each other, challenge one another’s reasoning, provide constructive feedback and accept other’s perspective [35]. A significant improvement in interpersonal skill of the students was noticed following the intervention which made them realise that their peers were knowledgeable, dependable, and trustworthy. Mizuno [42] stated that Cooperative Learning creates a learning environment where students can ask, clarify their ideas as well as develop respect for each other. It provides students an opportunity to know each other better and manage their relationship with the group members effectively [43]. Besides, imitating their peers in the group and receiving immediate feedback on their practiced social skills helps [17].

An enhancement in students’ ability to listen, respect other’s ideas and clearly express their point of view reflected an improvement in communication skill after JCL sessions. Students perceived these sessions boosted their confidence, presentation skills and the shy students overcame their hesitation which echo remarks made by previous researchers [14, 26, 37]. The students learnt appropriate ways to react to criticism during CL sessions [43]. The ability to get along with peers, share the task and work cooperatively to achieve the common goal were observed after JCL sessions. The participants of the study realised that listening to other’s opinion would facilitate the decision making without any conflicts, arguments, or fights. Gonzales & Torres [25] agree that Cooperative Learning helps students appreciate each other’s contribution during the group activity and facilitates their ability to build consensus.

The agencies of higher education, accrediting and the certifying bodies recommends outcome-based environments facilitating core academic subject mastery and skill development (collaboration, communication, critical thinking, and creativity) [4, 5]. One of the strategies which can be used to achieve these twenty-first century skills would be Jigsaw Cooperative Learning (JCL). This present study findings were based on multiple sessions of JCL over one whole semester giving students ample time to develop these skills and share their reflection on the impact of JCL. Educators can therefore use this learning strategy in their toolkit to promote the development of various core competencies expected of health professionals such as interpersonal, communication, collaborative, and teamwork skills. The abilities and skills learnt during JCL is useful even in interprofessional education and interprofessional practice. Sim et al. [44] found this learning technique ignites stronger culture of cooperation among students during interprofessional learning. Further, each group represents a miniature of the community. During the sessions, teachers teach the learners to create, monitor and evaluate the equity in the cooperative group. Thereby the students learn to create a just society.

This study has some limitations. The findings were based on the observations in a single medical school. Besides, the study did not include a control group, which was not possible for ethical reasons. Also, the first author is a teaching faculty for the study population, however, the participants were reassured that participation or denial will not impact them in any way.

## Conclusion

A Jigsaw Cooperative Learning technique significantly improves communication skill, interpersonal skill, critical thinking, interdependence, accountability, promotive interaction, and consensus building among students. It effectively trains students as responsible collaborators for the future healthcare teams. The educators and policy makers should consider introducing Jigsaw Cooperative Learning during the early years of training.

## Data Availability

Substantial amount of data generated or analysed during this study has been included in this published article. The datasets generated and/or analysed during the current study are not publicly available due to ethical guidelines and institutional regulations. However, these can be provided by the first author [AKJ] upon reasonable request.
